# International multicentre validation of the left pancreatectomy pancreatic fistula prediction models and development and validation of the combined DISPAIR-FRS prediction model

**DOI:** 10.1093/bjs/znae313

**Published:** 2025-03-21

**Authors:** Akseli Bonsdorff, Trond Kjeseth, Jakob Kirkegård, Charles de Ponthaud, Poya Ghorbani, Johanna Wennerblom, Caroline Williamson, Alexandra W Acher, Manoj Thillai, Timo Tarvainen, Ilkka Helanterä, Aki Uutela, Jukka Sirén, Arto Kokkola, Mushegh Sahakyan, Dyre Kleive, Rolf Hagen, Andrea Lund, Mette F Nielsen, Jean-Christophe Vaillant, Richard Fristedt, Christina Biörserud, Svein O Bratlie, Bobby Tingstedt, Knut J Labori, Sébastien Gaujoux, Stephen J Wigmore, Julie Hallet, Ernesto Sparrelid, Ville Sallinen

**Affiliations:** Department of Gastroenterological Surgery, Helsinki University Hospital and University of Helsinki, Helsinki, Finland; Department of Hepato-Pancreato-Biliary Surgery, Rikshospitalet, Oslo University Hospital, Oslo, Norway; Department of Clinical Medicine, University of Bergen, Bergen, Norway; Department of Surgery, HPB Section and Institute for Clinical Medicine, Aarhus University Hospital, Aarhus University, Aarhus, Denmark; Department of Digestive, Hepato-Pancreato-Biliary Surgery and Liver Transplantation, AP-HP Pitié-Salpêtrière Hospital, Sorbonne Université Paris, Paris, France; Division of Surgery and Oncology, Department of Clinical Science, Intervention, and Technology, Karolinska Institutet, Karolinska University Hospital, Stockholm, Sweden; Department of Surgery, Sahlgrenska University Hospital, Gothenburg, Sweden; Department of Surgery, Skåne University Hospital at Lund, Lund University, Lund, Sweden; Department of Surgery, University of Toronto and Sunnybrook Health Sciences Centre, Toronto, Ontario, Canada; Hepatobiliary and Pancreatic Unit & Edinburgh Transplant Unit, University of Edinburgh, Royal Infirmary, Edinburgh, UK; Department of Gastroenterological Surgery, Helsinki University Hospital and University of Helsinki, Helsinki, Finland; Department of Transplantation and Liver Surgery, Helsinki University Hospital and University of Helsinki, Helsinki, Finland; Hepatobiliary and Pancreatic Unit & Edinburgh Transplant Unit, University of Edinburgh, Royal Infirmary, Edinburgh, UK; Department of Transplantation and Liver Surgery, Helsinki University Hospital and University of Helsinki, Helsinki, Finland; Department of Gastroenterological Surgery, Helsinki University Hospital and University of Helsinki, Helsinki, Finland; Department of Gastroenterological Surgery, Helsinki University Hospital and University of Helsinki, Helsinki, Finland; Department of Surgery, Vestre Viken Hospital Trust, Ringerike Hospital, Hønefoss, Norway; The Intervention Centre, Oslo University Hospital, Rikshospitalet, Oslo, Norway; Department of Surgery N1, Yerevan State Medical University, Yerevan, Armenia; Department of Hepato-Pancreato-Biliary Surgery, Rikshospitalet, Oslo University Hospital, Oslo, Norway; Department of Surgery, Vestfold Hospital Trust, Tønsberg, Norway; Department of Surgery, HPB Section and Institute for Clinical Medicine, Aarhus University Hospital, Aarhus University, Aarhus, Denmark; Department of Surgery, HPB Section and Institute for Clinical Medicine, Aarhus University Hospital, Aarhus University, Aarhus, Denmark; Division of Surgery and Oncology, Department of Clinical Science, Intervention, and Technology, Karolinska Institutet, Karolinska University Hospital, Stockholm, Sweden; Department of Surgery, Skåne University Hospital at Lund, Lund University, Lund, Sweden; Department of Surgery, Sahlgrenska University Hospital, Gothenburg, Sweden; Department of Surgery, Sahlgrenska University Hospital, Gothenburg, Sweden; Department of Surgery, Skåne University Hospital at Lund, Lund University, Lund, Sweden; Department of Hepato-Pancreato-Biliary Surgery, Rikshospitalet, Oslo University Hospital, Oslo, Norway; Institute of Clinical Medicine, University of Oslo, Oslo, Norway; Division of Surgery and Oncology, Department of Clinical Science, Intervention, and Technology, Karolinska Institutet, Karolinska University Hospital, Stockholm, Sweden; Hepatobiliary and Pancreatic Unit & Edinburgh Transplant Unit, University of Edinburgh, Royal Infirmary, Edinburgh, UK; Department of Surgery, University of Toronto and Sunnybrook Health Sciences Centre, Toronto, Ontario, Canada; Department of Digestive, Hepato-Pancreato-Biliary Surgery and Liver Transplantation, AP-HP Pitié-Salpêtrière Hospital, Sorbonne Université Paris, Paris, France; Department of Gastroenterological Surgery, Helsinki University Hospital and University of Helsinki, Helsinki, Finland; Department of Transplantation and Liver Surgery, Helsinki University Hospital and University of Helsinki, Helsinki, Finland

## Abstract

**Background:**

Every fifth patient undergoing left pancreatectomy develops a postoperative pancreatic fistula (POPF). Accurate POPF risk prediction could help. Two independent preoperative prediction models have been developed and externally validated: DISPAIR and D-FRS. The aim of this study was to validate, compare, and possibly update the models.

**Methods:**

Patients from nine high-volume pancreatic surgery centres (8 in Europe and 1 in North America) were included in this retrospective cohort study. Inclusion criteria were age over 18 years and open or minimally invasive left pancreatectomy since 2010. Model performance was assessed with discrimination (receiver operating characteristic (ROC) curves) and calibration (calibration plots). The updated model was developed with logistic regression and internally-externally validated.

**Results:**

Of 2284 patients included, 497 (21.8%) developed POPF. Both DISPAIR (area under the ROC curve (AUC) 0.62) and D-FRS (AUC 0.62) performed suboptimally, both in the pooled validation cohort combining every centre’s data and centre-wise. An updated model, named DISPAIR-FRS, was constructed by combining the most stable predictors from the existing models and incorporating other readily available patient demographics, such as age, sex, transection site, pancreatic thickness at the transection site, and main pancreatic duct diameter at the transection site. Internal-external validation demonstrated an AUC of 0.72, a calibration slope of 0.93, and an intercept of −0.02 for the updated model.

**Conclusion:**

The combined updated model of DISPAIR and D-FRS named DISPAIR-FRS demonstrated better performance and can be accessed at www.tinyurl.com/the-dispair-frs.

## Teaser

Postoperative pancreatic fistula is a serious and dreaded complication after distal pancreatectomy, with an average incidence of 20%. Its preoperative risk estimation could help clinicians with decision-making and researchers with study planning. Neither of the existing prediction models—DISPAIR and D-FRS—for pancreatic fistula had stable predictions, presumably due to unreliable predictor modelling in the original studies. A combinatory model, named DISPAIR-FRS, utilizing the most stable predictors from both of the models seems to have better performance and could offer better generalizability and transportability.

## Introduction

The clinical significance of postoperative pancreatic fistula (POPF) after pancreatic resections is undisputed. It not only causes morbidity and increased length of hospitalization and costs, but is also associated with increased postoperative mortality^1^. Therefore, it is imperative that future studies focus on its effective prevention and treatment. Better identification of individual POPF risk could help clinicians, patients, and clinical researchers with decision-making, treatment allocation, and study planning.

Patient-specific factors, such as pancreatic texture, age, and BMI, modify the risk of POPF^2,3^. For patients undergoing distal pancreatectomy, there are two externally validated POPF prediction models, both published in 2022, DISPAIR and D-FRS^4,5^. Both models use pancreatic anthropomorphic measurement from preoperative CT images, such as pancreatic thickness (PT) and main pancreatic duct diameter (MPD). As the prediction models are already being implemented in large scale trials, it is essential their performance is assessed thoroughly^6^.

The aim of this international multicentre study was to assess and compare the stability and feasibility of the modelled predictors and to explore the possibility of combining the existing models into a better performing one.

## Methods

The TRIPOD checklist was utilized in the reporting of this study (*Table S1*)^7^. A recent consensus paper underlined the existing heterogeneity in what constitutes distal pancreatectomy and proposed a new classification based on the extensiveness of pancreatic resection and shifting to using the term left pancreatectomy^8^. For this reason, left pancreatectomy was used instead of distal pancreatectomy.

### Participants

This was an international multicentre retrospective study. A total of nine high-volume (>10 left pancreatectomies/year) pancreatic surgery centres (8 in Europe and 1 in North America) provided data for consecutive patients undergoing left pancreatectomy after 1 January 2010^9^. Inclusion criteria were age over 18 years and open or minimally invasive left pancreatectomy with or without splenectomy. Each participating centre had to be able to include at least 150 patients. Patient data were obtained using medical record review by each participating centre according to their local regulations and legislation, anonymized, and sent electronically to the coordinating team using encryption and password protection. The study had institutional review board approval from each of the participating centres.

### Sample size

Due to the retrospective setting, available data in the participating centres dictated the sample size. The number of predictor parameters that could be used in model development given the available sample was calculated with the pmsampsize package in R^10^. The assumptions made were an expected Nagelkerke R^2^ of 0.15 and 20% POPF prevalence.

### Outcome

The main outcome was POPF (grades B and C) as defined in 2016 by the International Study Group for Pancreatic Surgery (ISGPS)^11^.

### Predictors

Both DISPAIR and D-FRS use variables measured from axial sections of the most recent preoperative CT images that are available before surgery^4,5^. Each participating centre performed the measurements retrospectively according to a guide provided by the coordinating team (*Fig. S1*). History of diabetes was defined as being on any anti-diabetic medication.

The MPD at the pancreatic neck (MPD-n) and the MPD at the transection site (MPD-t) were measured and rounded to the nearest 1 mm. If the duct was not visible in CT images, then 1.0 mm was entered. The PT at the neck (PT-n) and the PT at the transection site (PT-t) were also measured. The transection site was retrospectively determined with the help of postoperative CT images, the resected specimen length from the pathologist’s report, or operation notes. In a real-life setting, the transection site would be decided before surgery by the operating surgeon and measurements would be performed at that site. For DISPAIR validation, the transection site was dichotomized at the left border of the superior mesenteric vein in line with the recent consensus paper^8^. Transection left of this landmark was defined as ‘body/tail’ and otherwise ‘head/neck’. For model updating, the transection site was treated as a three-category variable: head *versus* neck *versus* body/tail. Transection at the head was differentiated from transection at the neck by happening right of the gastroduodenal artery. MPD and PT were used in analyses as continuous variables and, as logistic regression assumes a linear association between the logit of the outcome and the predictor, their graphical associations were assessed with restricted cubic splines with three and four knots^12^.

MPD and PT values were missing for some patients because of missing or unavailable preoperative CT images. These were considered to be missing at random and values were imputed using multiple imputation with fully conditional specification with ten iterations using the mice package in R^13^. Missing values for POPF and BMI were also similarly imputed.

### DISPAIR and D-FRS equations

Specifically, in this study, preoperative D-FRS was assessed. The following regression model linear predictors were used for model validation:


LP(D-FRS)=−4.211+0.388[MPD-n]+0.131[PT-n]

LP(DISPAIR)=−8.322+0.384[PT-t]+0.545[Transectionathead/neck]−1.116[Diabetes]

Predicted probability was calculated as $P=\frac{e^{LP}}{1+e^{LP}}$.

### Model validation

External model validity was assessed with overall model fit, discrimination, and calibration plots^14^. D-FRS was validated in the whole data set and DISPAIR was validated in a cohort omitting data from Helsinki, as the model was originally developed in that data set. Overall model fit was measured with Nagelkerke R^2^. Discrimination was the model’s ability to classify patients with or without the outcome and is reported as an area under the receiver operating characteristic (ROC) curve (AUC) value, with 95% confidence interval. Discrimination did not consider the relative accuracy of predictions (that is how close the predicted probabilities were to the observed probabilities) and thus calibration plots were required. The most important measures of calibration are the calibration intercept and slope. The intercept measures the difference between average predicted and average observed probabilities and has a target value of zero (no difference). The slope measures the spread of predicted probabilities and has a target value of one. Extreme predictions (too high for high-risk patients and too low for low-risk patients) are often a sign of overfit during model development and result in calibration slope values less than one^15^. Conversely, predictions that are too modest (too low for high-risk patients and too high for low-risk patients) result in calibration slope values greater than one.

### Model updating and internal-external validation

The updated model was created with logistic regression by including the predictors used in DISPAIR and D-FRS, and readily available patient demographics. Historical institutional POPF incidence was used as a variable to better calibrate the predictions for different centres. It was categorized at 15% and 25% to acquire similar sized groups and because the average reported POPF incidence in the literature was approximately 20%^4,5,16^. Internal-external validation was commenced by combining participating centres into clusters of similar size. Helsinki and Oslo formed cluster one, all Swedish centres (Stockholm, Lund, and Gothenburg) formed cluster two, and the rest of the centres (Aarhus, Paris, Edinburgh, and Toronto) formed cluster 3. By clustering, a sufficient sample size for validation was ensured, while retaining geographical diversity. The model was fitted in all possible combinations of clusters, retaining one cluster for validation each time (for example, the model was fitted in a cohort combining clusters 1 and 2, and validated in cluster 3). Model performance is reported both cluster-wise and as a weighted average. The final model regression coefficients were obtained by fitting the internally-externally validated model in the whole data set^17^. The goal with internal-external validation was to test and show the stability of model predictions, while using all the available data for estimating model coefficients^17,18^. After acquiring the final model, sensitivity analyses were performed to assess its performance in different subgroups. These subgroups were defined by transection site, operation type (minimally invasive or open), perioperative intra-abdominal drainage, prophylactic somatostatin analogue (pasireotide or octreotide), obesity, and stump closure method (stapler *versus* handsewn). Decision curve analysis (DCA) by bootstrapping was performed with the rmda package in R to assess the potential net benefit of using the prediction models in a clinical setting. DCA was used to assess the potential net benefit (how many more true positives would be allocated to an intervention if the model was used for stratification) over different prediction thresholds compared with two extreme alternatives: intervention for all and intervention for none^19,20^.

### Statistics

All continuous variables are reported as median (interquartile range). Categorical variables are reported as *n* (%). Statistical significance between groups was assessed with the chi-squared test for categorical variables and the Mann–Whitney *U* test or Kruskal–Wallis test for continuous variables. Potential multicollinearity was assessed with variance inflation factors. Partial predictor effect plots are used to show the relationship of single predictors in a prediction model, while adjusting for interference from other predictor variables. All analyses were performed with R (R Core Team. *R: A Language and Environment for Statistical Computing*. Vienna: R Foundation for Statistical Computing, 2023; https://www.R-project.org/) in RStudio. The following R packages were used: rms, rmda, ggplot2, Hmisc, DescTools, forestplot, mice, and pmsampsize. The two-sided significance level was set at 0.05.

## Results

In total, 2284 patients were included in the study with the sample size per participating centre ranging between 151 (6.6%) and 538 (23.6%) (*Table 1* and *Table S2*). The overall POPF incidence was 497 (21.8%), ranging from 8.0% to 39.3% between centres. There were some differences regarding perioperative protocols between the participating centres, such as perioperative intra-abdominal drainage protocols, method of transection, transection site, and administration of somatostatin analogues (*Table S2*). The study flow chart is presented in *Fig. S2*.

**Table 1:** Patient demographics, perioperative details, and postoperative outcomes in the total cohort of 2284 patients undergoing left pancreatectomy; complete case analysis

	Total	Missing values
**Time span**	January 2010 to April 2023	0
**Age (years), median (i.q.r.)**	65 (53–72)	0
**Male sex**	1059 (46.3)	0
**BMI (kg/m^2^), median (i.q.r)**	25.9 (23.0–29.1)	67 (2.9)
**History of myocardial infarction**	130 (5.7)	2 (0.1)
**History of COPD**	224 (9.8)	2 (0.1)
**History of diabetes**	441 (19.3)	1 (0.04)
**Neoadjuvant therapy (any)**	139 (6.1)	0
**Operation type**		
Open	1156 (50.6)	3 (0.1)
Laparoscopic	1046 (45.8)	
Robotic	81 (3.5)	
**Transection method**		
Stapler	1980 (86.7)	6 (0.3)
Handsewn	268 (11.7)	
Other	30 (1.3)	
**Transection site**		
Head	67 (2.9)	182 (8.0)
Neck	967 (42.3)	
Body/tail	1068 (46.8)	
**Use of somatostatin analogue**		
None	1627 (71.2)	152 (6.6)
Prophylactic	367 (16.1)	
Treatment	140 (6.1)	
**Perioperative intra-abdominal drainage**	1990 (87.1)	3 (0.1)
**Initial length of stay (days), median (i.q.r.)**	7 (5–11)	14 (0.6)
**Readmission**	344 (15.0)	47 (2.1)
**Ninety-day mortality**	31 (1.4)	153 (6.7)
**POPF (grade B/C) up to 30 days after surgery**	497 (21.8)	0
**Tumour histology**		
PDAC	651 (28.5)	5 (0.2)
IPMN	296 (13.0)	
NET	584 (25.6)	
Other malign	192 (8.4)	
Other benign	505 (22.1)	
Dysplasia	16 (0.7)	
Not diagnostic	37 (1.6)	

Values are *n* (%) unless otherwise indicated. i.q.r., interquartile range; COPD, chronic obstructive pulmonary disease; POPF, postoperative pancreatic fistula; PDAC, pancreatic ductal adenocarcinoma; IPMN, intraductal papillary mucinous neoplasm; NET, neuroendocrine tumour.

### External validation of DISPAIR and D-FRS

**Fig. 1:**
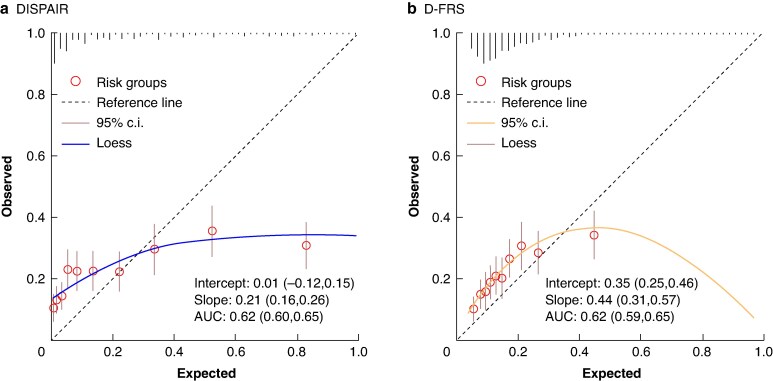
Calibration plots for DISPAIR and D-FRS in the total cohort of 2018 and 2284 patients undergoing left pancreatectomy respectively **a** DISPAIR. **b** D-FRS. The expected (predicted) POPF probability is shown on the *x*-axis and the observed (actual) POPF probability is shown on the *y*-axis. Circles represent deciles (10% of patients) and vertical lines represent distribution of predictions (histogram). AUC, area under the receiver operating characteristic curve; POPF, postoperative pancreatic fistula.

The overall fit was poor for both models (Nagelkerke R^2^ 0.035 and 0.021 for DISPAIR and D-FRS respectively). Both models demonstrated similar discrimination. DISPAIR had an AUC of 0.62 (95% c.i. 0.60 to 0.65) and D-FRS had an AUC of 0.62 (95% c.i. 0.59 to 0.65). DISPAIR had a calibration intercept of 0.01 (95% c.i. −0.12 to 0.15) and a calibration slope of 0.21 (95% c.i. 0.16 to 0.26) and D-FRS had a calibration intercept of 0.35 (95% c.i. 0.25 to 0.46) and a calibration slope of 0.44 (95% c.i. 0.31 to 0.57). Calibration plots show that both models tend to significantly overestimate the probability of POPF at >20% predictions (*Fig. 1*). The performance of each model was also assessed centre-wise, but neither demonstrated good performance (*Figs S3*, *S4*).

### Predictor diagnostics

Predictors used in DISPAIR and D-FRS were further assessed for their association with POPF in the whole cohort. The associations between PT and MPD measurements and POPF were fairly linear and no transformations were deemed necessary (*Fig. S5a–d*).

Unadjusted ORs for POPF were calculated for all four pancreatic measurements: MPD-n, 1.04 (95% c.i. 0.95 to 1.13); MPD-t, 0.97 (95% c.i. 0.88 to 1.05); PT-n, 1.12 (95% c.i. 1.10 to 1.15); and PT-t, 1.10 (95% c.i. 1.08 to 1.12); all reported per mm of increase. The unadjusted OR was 0.96 (95% c.i. 0.74 to 1.23) for history of diabetes and 1.51 (95% c.i. 1.24 to 1.85) for transection at the pancreatic head/neck. Centre-wise associations are presented as forest plots (*Fig. S6*)^4,5^.

### Model updating and creation of a new prediction model

Model updating was commenced, as both models seemed to contain significant overfit and non-stable modelling of some predictors. In addition to the predictors used in DISPAIR and D-FRS, patient age, BMI, sex, and neoadjuvant therapy were included as candidate predictors because of their preoperative availability and previous evidence regarding possible association with POPF^21–29^. Age showed a non-linear association with POPF and was modelled with splines with three knots, as modelling with four knots did not significantly differ (*Fig. S5e*). Transection site was incorporated into the updated model as a three-category variable (head *versus* neck *versus* body/tail).

According to the sample size calculation, 25 predictor parameters could be included in model development. *Table 2* shows the variable elimination process and final coefficients for the updated model. PT-t and MPD-t outperformed corresponding measurements at the neck and, due to potential multicollinearity, PT-n and MPD-n were excluded from the model. The final updated model contains six predictor parameters: PT-t (linear); MPD-t (linear); age (non-linear); sex (categorical); and transection at head *versus* neck *versus* body/tail (categorical).

**Table 2:** Predictor elimination process and development of DISPAIR-FRS

Predictor	Reason for inclusion	All candidate predictors		Elimination (reason)	DISPAIR-FRS, OR (95% c.i.)	DISPAIR-FRS with historical POPF incidence, OR (95% c.i.)
		OR (95% c.i.)	VIF			
**PT-n (5–37 mm)**	In D-FRS	1.02 (0.98,1.05)	1.61	Yes (PT-t stronger and multicollinearity)		
**PT-t (5–40 mm)**	In DISPAIR	1.11 (1.08,1.13) per mm	1.76	No	1.12 (1.10,1.14) per mm	1.12 (1.09,1.14)
**MPD-n (1–8 mm)**	In D-FRS	1.14 (0.98,1.32)	2.69	Yes (MPD-t stronger and multicollinearity)		
**MPD-t (1–8 mm)**	Similar to MPD-n	0.82 (0.70,0.97)	2.75	No	0.96 (0.87,1.05)	0.92 (0.83,1.01)
**Transection site**						
Body/tail	In DISPAIR	Reference		No	Reference	Reference
Neck		1.49 (1.14,1.94	1.57		2.45 (1.94,3.09)	1.55 (1.20,2.00)
Head		1.67 (0.92,2.95)	1.19		3.45 (2.02,5.80)	1.71 (0.96,3.00)
**Age (non-linear)**	Reported in the literature^24^	NA (see *Fig. 2*)		No	Non-linear*	Non-linear*
**BMI (linear)**	Reported in the literature^22,25,26^	1.01 (0.99,1.03)	1.11	Yes (non-siginificant association)		
**Male sex**	Reported in the literature^23,29^	1.20 (0.97,1.50)	1.04	No	1.26 (1.02,1.56)	1.22 (0.98,1.51)
**Neoadjuvant therapy**	Reported in the literature^27,28^	0.93 (0.58,1.46)	1.02	Yes (non-significant association)		
**Historical POPF incidence**						
<15%		Reference		No		Reference
15–25%		2.29 (1.70,3.12)				2.32 (1.73,3.16)
>25%		5.73 (4.12,8.04)				5.79 (4.18,8.12)
**Apparent performance**						
Apparent AUC		0.74 (0.72,0.77)			0.69 (0.66,0.72)	0.74 (0.72,0.77)
Apparent Nagelkerke R^2^		0.198			0.121	0.195

^*^Age was modelled with restricted cubic splines with three knots and no ORs can be reported for them. Please see *Fig. 2* for predictor effects. VIF, variance inflation factor; POPF, postoperative pancreatic fistula; PT-n, pancreatic thickness at the neck; PT-t, pancreatic thickness at the transection site; MPD-n, main pancreatic duct diameter at the neck; MPD-t; main pancreatic duct diameter at the transection site; NA, not applicable; AUC, area under the receiver operating characteristic curve.

**Fig. 2:**
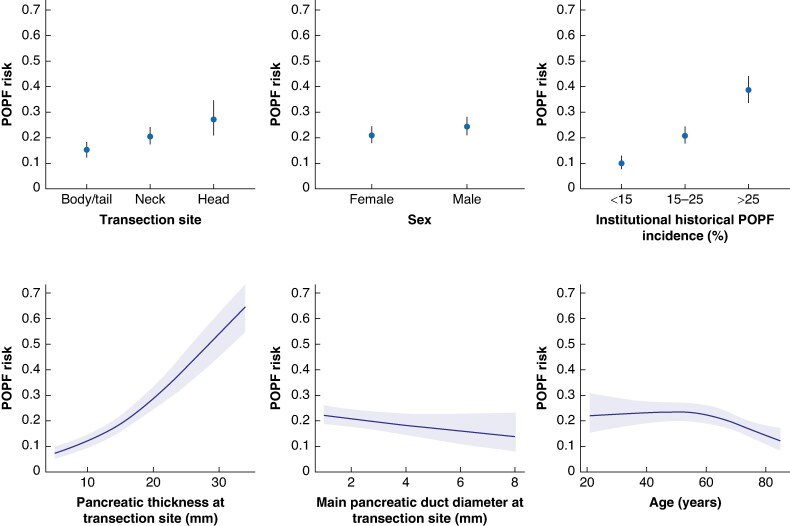
Partial effect plots for each DISPAIR-FRS predictor The predictor value is shown on the *x*-axis and the POPF probability is shown on the *y*-axis. POPF, postoperative pancreatic fistula.

The model was fitted both with and without historical POPF incidence (<15%, 4 centres (720 patients); 15–25%, 3 centres (989 patients); and >25%, 2 centres (575 patients)), as the option of using only patient-specific predictors was warranted. The model had an apparent AUC value of 0.69 (95% c.i. 0.66 to 0.72) when omitting historical POPF incidence and 0.74 (95% c.i. 0.72 to 0.77) when incorporating historical POPF incidence. The non-linear predictor age had a protective association with POPF, especially for patients over 50 years of age (*Fig. 2*). A nomogram is also presented for easier model interpretation (*Fig. S7*). As the new model incorporated parameters from DISPAIR and D-FRS, it was named DISPAIR-FRS.

### Validation of DISPAIR-FRS

**Fig. 3:**
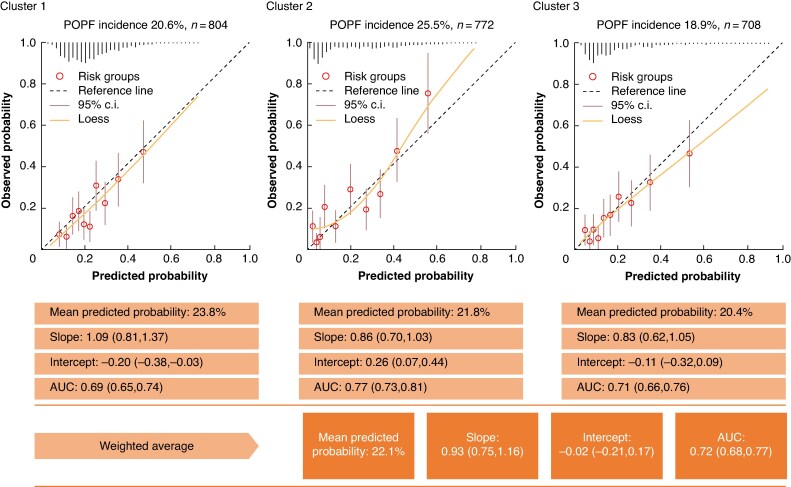
Results of internal-external validation of the full DISPAIR-FRS prediction model in a cohort of 2284 patients undergoing left pancreatectomy For each iteration, one cluster was left out for validation and the model was fitted in the remaining clusters. In calibration plots, circles represent deciles (10% of patients) and vertical lines represent distribution of predictions (histogram). Centres in cluster 1: Helsinki and Oslo. Centres in cluster 2: Stockholm, Lund, and Gothenburg. Centres in cluster 3: Aarhus, Paris, Edinburgh, and Toronto. POPF, postoperative pancreatic fistula; AUC, area under the receiver operating characteristic curve.

Internal-external validation was performed (*Fig. 3* and *Fig. S8*). When omitting historical POPF incidence from the model, the weighted average AUC was 0.66 (95% c.i. 0.62 to 0.71), the calibration slope was 0.84 (95% c.i. 0.59 to 1.09), and the calibration intercept was 0.04 (95% c.i. −0.14 to 0.22). Likewise, for the full model, the weighted average AUC was 0.72 (95% c.i. 0.68 to 0.77), the calibration slope was 0.93 (95% c.i. 0.75 to 1.16), and the calibration intercept was −0.02 (95% c.i. −0.21 to 0.17). A histogram of predicted probabilities stratified by POPF is presented in *Fig. S9*.

The final DISPAIR-FRS model equations are presented in the *supplementary results*. The DISPAIR-FRS web-based calculator can be found at www.tinyurl.com/the-dispair-frs.

### Sensitivity analyses

The performance of the full DISPAIR-FRS model was assessed in different subgroups to assess its generalizability to different populations (*Fig. S10*). The performance of DISPAIR-FRS remained similar in all of the different subgroups, except a subgroup of patients who did not undergo perioperative intra-abdominal drainage (291 patients). Its discrimination and spread of predictions were satisfactory (AUC 0.66 and calibration slope 0.94), but there was significant systematic POPF risk overestimation in this group (calibration intercept −0.74).

### Risk groups and net benefit

Three risk groups (low, <10%; moderate, 10–30%; and high, >30%) were formed to demonstrate the distribution of different clinical outcomes at different DISPAIR-FRS levels (*Table S3*). POPF incidence increased from 8.0% (50 of 627) in the low-risk group to 17.4% (185 of 1064) in the moderate-risk group and to 44.2% (262 of 593) in the high-risk group (*P* < 0.001). Differences were statistically significant for grade B and C POPF, postpancreatectomy haemorrhage, reoperation, readmission, and length of initial hospital stay. Only 90-day mortality remained similar in all of the risk groups.

DCA demonstrated potential net benefit over the whole spectrum of DISPAIR-FRS predictions in general and when compared with the other models (*Fig. S11*). For example, changing management at a 20% risk threshold (for example patient selection or mitigation strategies for POPF), using DISPAIR-FRS could potentially lead to a net benefit of 0.07 (detecting 7 more true positives per 100 patients) and lead to a reduction of 25 unnecessary interventions (for example mitigation/prophylactic measures) per 100 patients.

## Discussion

The validity of two previously published prediction models, DISPAIR and D-FRS, demonstrated underwhelming external performance. The models were also miscalibrated, especially with systematic risk overestimation at over 20% predicted risk. In their current state, neither of the models should be applied in clinical settings, as miscalibrated predictions might result in misguided decision-making. However, the updated model—named DISPAIR-FRS—combining predictors from the existing models and re-estimating the coefficients, showed stability and superior performance and net benefit in DCA.

Many prediction models for POPF have been published during the recent decade, albeit predominantly for pancreatoduodenectomies^30^. While the original studies of DISPAIR and D-FRS stand out from the bulk in a methodological sense, they still fell short in terms of generalizability. AUC values in the original studies were 0.80 (95% c.i. 0.75 to 0.85) for DISPAIR and 0.73 (95% c.i. 0.70 to 0.76) for D-FRS. According to their external calibration slopes in the present study, a significant amount of overfit was presumably modelled during original model development. This might have stemmed from relatively small sample sizes (266 for DISPAIR and 339 for D-FRS)^10^. Just recently, a Dutch nationwide validation of preoperative D-FRS with 896 patients was performed, reporting an AUC of 0.73 (95% c.i. 0.68 to 0.78)^16^. Another recent validation study from China with 653 patients demonstrated that preoperative D-FRS and DISPAIR have similar AUC values (0.72 (95% c.i. 0.69 to 0.76) for both models)^31^. Why the models performed better in terms of discrimination in these cohorts compared with the cohort in the present study is challenging to assess, as neither of the studies reported or assessed partial predictor effects or calibration. Of note, even though POPF is well defined by the ISGPS, significant heterogeneity in POPF classification and diagnostics exists between centres, and maybe even to a larger degree between countries, due to different perioperative protocols^32^. Thus, it is of paramount importance to validate models outside of the country of origin.

According to the presented evidence, the modelling of history of diabetes in DISPAIR (OR 0.34) and the modelling of MPD-n in D-FRS (OR 1.46 per mm) were questionable and probably partially drove the miscalibrated predictions. In the present study, neither diabetes nor MPD-n had a noteworthy association with POPF, as demonstrated by their unadjusted ORs of 0.96 and 1.04 (per mm) respectively. Growing PT-n and PT-t, however, could be established as more reliable predictors of POPF through this study.

DISPAIR-FRS includes PT-t, MPD-t, age, sex, and transection site (head *versus* neck *versus* body/tail). To account for the large variation in POPF incidence, the historical institutional incidence was incorporated into the model. When using the model, the user has the option to either use only patient-specific predictors or incorporate institutional historical POPF incidence. The full model should be preferred in clinical work, as it has slightly more stable calibration and better discrimination. For research, benchmarking, or audit purposes, for example when assessing case mix between surgeons or centres, or the possible effect of a mitigation strategy for POPF, the model without historical POPF incidence should be used. Also, the patient-specific version allows use in settings where historical POPF incidence is not known.

Radiologically measured PT as a reliable risk factor for POPF is increasingly supported^33,34^. The authors hypothesize that a thicker pancreas translates to a more challenging stump closure, therewith increasing risk of leakage and ultimately POPF. Modelling PT in DISPAIR-FRS at the future transection site instead of the pancreatic neck also seems clinically more reasonable, as it better corresponds to the actual amount of tissue transected. It also allows for more dynamic predictions, as the user can simulate approaches with different transection levels (thus possibly different PT) to find an optimal site for transection in terms of POPF risk. Transecting at the head (OR 1.70) or neck (OR 1.50) are modelled as risk factors in DISPAIR-FRS, but the interplay between transection site and PT should be taken into account when interpreting the effect of single predictors. The pancreas is usually thicker in the body/tail area and thus more proximal transection might be more favourable in terms of POPF risk in some cases.

Age was modelled with splines, as it had a non-linear association with POPF. Its protective association was stronger for patients over 50 years of age. This might be explained by increasing levels of pancreatic fibrosis with ageing. Male sex is modelled as a moderate risk factor for POPF, which is concordant with previous studies^23,29^.

Limitations of the present study include its retrospective design, which reduced the reliability of the data and resulted in some missing values. One significant challenge from a prediction modelling perspective was the large variation between centres in outcome event incidence, mostly owing to heterogeneity regarding the definition of POPF. Because POPF is influenced by the intensity of a treatment strategy, institutional protocols affect its reported incidence, especially for grade B POPF, which might have significant overlap with biochemical leak POPF, when comparing different centres^11,32^. For example, in a recent multicentre RCT, a postoperative care algorithm, which advocated for liberal use of postoperative CT images and based initiation of antibiotic treatment or intra-abdominal drainage on CT image findings, showed a reduction in postoperative mortality, but an increase in POPF incidence^35^. Recently, inter-observer variability with regard to the current ISGPS POPF definition was high for pancreatoduodenectomy and the authors do not expect it to differ a lot for left pancreatectomy. This is a probable factor behind the considerable variation in POPF incidence between centres in the present study as well^36^.

Sensitivity analyses showed DISPAIR-FRS to be generalizable to many different subpopulations. So, despite the heterogeneity in clinical practices or care received, such as minimally invasive operation or prophylactic somatostatin administration, DISPAIR-FRS should give reliable predictions. Only in a subgroup of patients with no intra-abdominal drains did DISPAIR-FRS give predictions that were systematically too high. Selection bias is thought to be responsible for this miscalibration, but, as shown in the recent PANDORINA trial, a no-drain policy is safe and could reduce the rate of POPF in left pancreatectomy^6^. If a no-drain policy becomes the standard of care in the future, DISPAIR-FRS should be recalibrated to account for this change in baseline POPF risk. However, in the present study, DISPAIR-FRS still showed good discrimination and a good calibration slope in the no-drain subgroup. Therefore, simple intercept recalibration could be sufficient.

Next steps in the DISPAIR-FRS pipeline include model deployment. As POPF remains the main driver of morbidity, targeting patients at high risk with potential treatments and mitigation strategies—such as somatostatin analogues^37,38^ and targeted intra-abdominal drainage^6,39^—could offer ways to reduce its impact and thus RCTs stratifying by POPF risk are warranted. DISPAIR-FRS could also be used for audit and benchmarking, allowing adjustments for the case mix. Also, as the effect of transection site and its interplay with PT is modelled into DISPAIR-FRS, it could be used to help guide surgical decision-making before surgery and intraoperatively. Eventually, once established into clinical practice, DISPAIR-FRS could provide ways for shared decision-making and more personalized care.

## Supplementary Material

znae313_Supplementary_Data

## Data Availability

Due to the international multicentre setting of this study and varying regional and national legislations, no data are available for sharing.
