# Supplementary material for: International multicentre validation of the left pancreatectomy pancreatic fistula prediction models and development and validation of the combined DISPAIR-FRS prediction model
Source: Br J Surg. 2025 Mar 21;112(3):znae313. doi: 10.1093/bjs/znae313 (PMC11926329; doi:10.1093/bjs/znae313)
Supplement: znae313_Supplementary_Data [file znae313_supplementary_data.docx]

**International multicentre validation of the left pancreatectomy pancreatic fistula prediction models and development and validation of the combined DISPAIR-FRS prediction model**

Akseli Bonsdorff MD PhD^1^, Trond Kjeseth MD^2,3^, Jakob Kirkegård MD PhD^4^, Charles de Ponthaud MD^6^, Poya Ghorbani MD PhD^5^, Johanna Wennerblom MD PhD^7^, Caroline Williamson MD PhD^8^, Alexandra W Acher MD^9^, Manoj Thillai MD^10^, Timo Tarvainen MD^1^, Ilkka Helanterä MD PhD^16^, Aki Uutela MD^10,16^, Jukka Sirén MD PhD^1^, Arto Kokkola MD PhD^1^, Mushegh Sahakyan MD PhD^11,12,13^, Dyre Kleive MD PhD^2^, Rolf Hagen MD^14^, Andrea Lund MD^4^, Mette Fugleberg Nielsen MD^4^, Jean-Christophe Vaillant MD PhD^5^, Richard Fristedt MD PhD^8^, Christina Biörserud PhD^7^, Svein Olav Bratlie MD PhD^7^, Bobby Tingstedt MD PhD^8^, Knut J. Labori MD PhD^2,15^, Sébastien Gaujoux MD PhD^5^, Stephen Wigmore MD FRCSED^10^, Julie Hallet MD^9^, Ernesto Sparrelid MD PhD^6^, Ville Sallinen MD PhD^1,16^

^1^ Helsinki University Hospital and University of Helsinki, Department of Gastroenterological Surgery

^2^ Department of Hepato-Pancreato-Biliary Surgery, Rikshospitalet, Oslo University Hospital

^3^ Department of Clinical Medicine, University of Bergen, Bergen, Norway

^4^ Aarhus University Hospital, Department of Surgery, HPB section and Institute for Clinical Medicine, Aarhus University

^5^ Department of Digestive, Hepato-Pancreato-Biliary Surgery and Liver Transplantation, AP-HP Pitié-Salpêtrière Hospital, Sorbonne Université Paris, France

^6^ Division of Surgery and Oncology, Department of Clinical Science, Intervention, and Technology, Karolinska Institutet, Karolinska University Hospital, Stockholm, Sweden

^7^ Department of Surgery, Sahlgrenska University Hospital, Gothenburg, Sweden

^8^ Lund University, Department of Surgery, Skåne University Hospital at Lund

^9^ Department of Surgery, University of Toronto and Sunnybrook Health Sciences Centre, Toronto, Ontario, Canada

^10^ Hepatobiliary and Pancreatic Unit & Edinburgh Transplant Unit, University of Edinburgh, Royal Infirmary, EH16 4SA, UK

^11^ Department of Surgery, Vestre Viken Hospital Trust, Ringerike Hospital, Hønefoss, Norway.

^12^ The Intervention Centre, Oslo University Hospital, Rikshospitalet, Oslo, Norway.

^13^ Department of Surgery N1, Yerevan State Medical University, Yerevan, Armenia.

^14^ Department of Surgery, Vestfold Hospital Trust, Tønsberg Norway

^15^ Institute of Clinical Medicine, University of Oslo, Norway

^16^ Helsinki University Hospital and University of Helsinki, Department of Transplantation and Liver Surgery

**Corresponding author.** Ville Sallinen, Adjunct professor, Head of Department, MD, PhD, Helsinki University Hospital and University of Helsinki, [ville.sallinen@helsinki.fi](mailto:ville.sallinen@helsinki.fi) Haartmaninkatu 4, 00029 Helsinki, Finland.

**ORCID ID**: 0000-0001-5394-4169, **Twitter**: @villesallinen

**Supplementary Materials - Index**

| **Supplementary Results** |  |
| --- | --- |
| Supplementary results 1 | *page 3* |
| **Supplementary Figures and Tables** |  |
| Supplementary figure 1 | *page 4* |
| Supplementary figure 2 | *page 5* |
| Supplementary figure 3 | *page 6* |
| Supplementary figure 4 | *page 7* |
| Supplementary figure 5 | *page 8* |
| Supplementary figure 6 | *page 9* |
| Supplementary figure 7 | *page 10* |
| Supplementary figure 8 | *page 11* |
| Supplementary figure 9 | *page 12* |
| Supplementary figure 10 | *page 13-14* |
| Supplementary figure 11 | *page 15* |
| Supplementary table 1 | *page 16-17* |
| Supplementary table 2 | *page 18* |
| Supplementary table 3 | *page 19* |

**Supplementary Results**

**Supplementary results 1**

Full DISPAIR-FRS model (for clinical use):

$$LP= -4.220 + 0.111[PT at transection site(mm)] + 0.00326[Age (years)] - 1.489e^{-5}*{pmax\left( age-41,0 \right)}^{3} + 4.468e^{-5}*{pmax\left( age-65,0 \right)}^{3}- 2.979e^{-5}*{pmax\left( age-77,0 \right)}^{3}+0.437[Transection at neck] + 0.539[Transection at head]-0.0838\left[ MPD at transection site \left( mm \right) \right]+0.197\left[ Male sex \right]+0.842\left[ Historical POPF \%: 15-25\% \right]+1.757[Historical POPF \%: >25\%]$$

DISPAIR-FRS model when omitting historical POPF incidence (using only patient specific predictors to be used for research, bechmarking, and audit purposes):

$$LP = -3.587 + 0.115\left[ PT at transection site\left( mm \right) \right]+ 0.000932\left[ Age \left( years \right) \right]- 1.316e^{-5}*{pmax\left( age-41,0 \right)}^{3} + 3.948e^{-5}*{pmax\left( age-65,0 \right)}^{3}- 2.632e^{-5}*{pmax\left( age-77,0 \right)}^{3}+0.894\left[ Transection at neck \right]+1.240[Transection at head]-0.0407\left[ MPD at transection site \left( mm \right) \right]+0.230\left[ Male sex \right]$$

Pmax-function in R is used to model spline knots for age, it selects the larger of the values divided by the comma, for example if age is 50, pmax(age-41,0) = pmax(50-41,0) = pmax(9,0) = 9.

PT-t is entered in mm, age in years, categorical variables as 1 if present. Predicted probability can be calculated as $P\left( \text{POPF} \right)=\frac{e^{LP}}{1+e^{LP}}$.

**Supplementary Figures and Tables**

**Supplementary figure 1.** Guide for doing preoperative CT-scan measurements retrospectively. **A)** Pancreatic thickness at neck (dark red line)**, B)** pancreatic thickness at transection site (red line). Transection site established with postoperative CT-scan (orange dashed line), pancreatic areas for defining transection site are presented (orange = head, blue = neck, red = body/tail, and **C)** transection site established with help of the pathologist’s report.


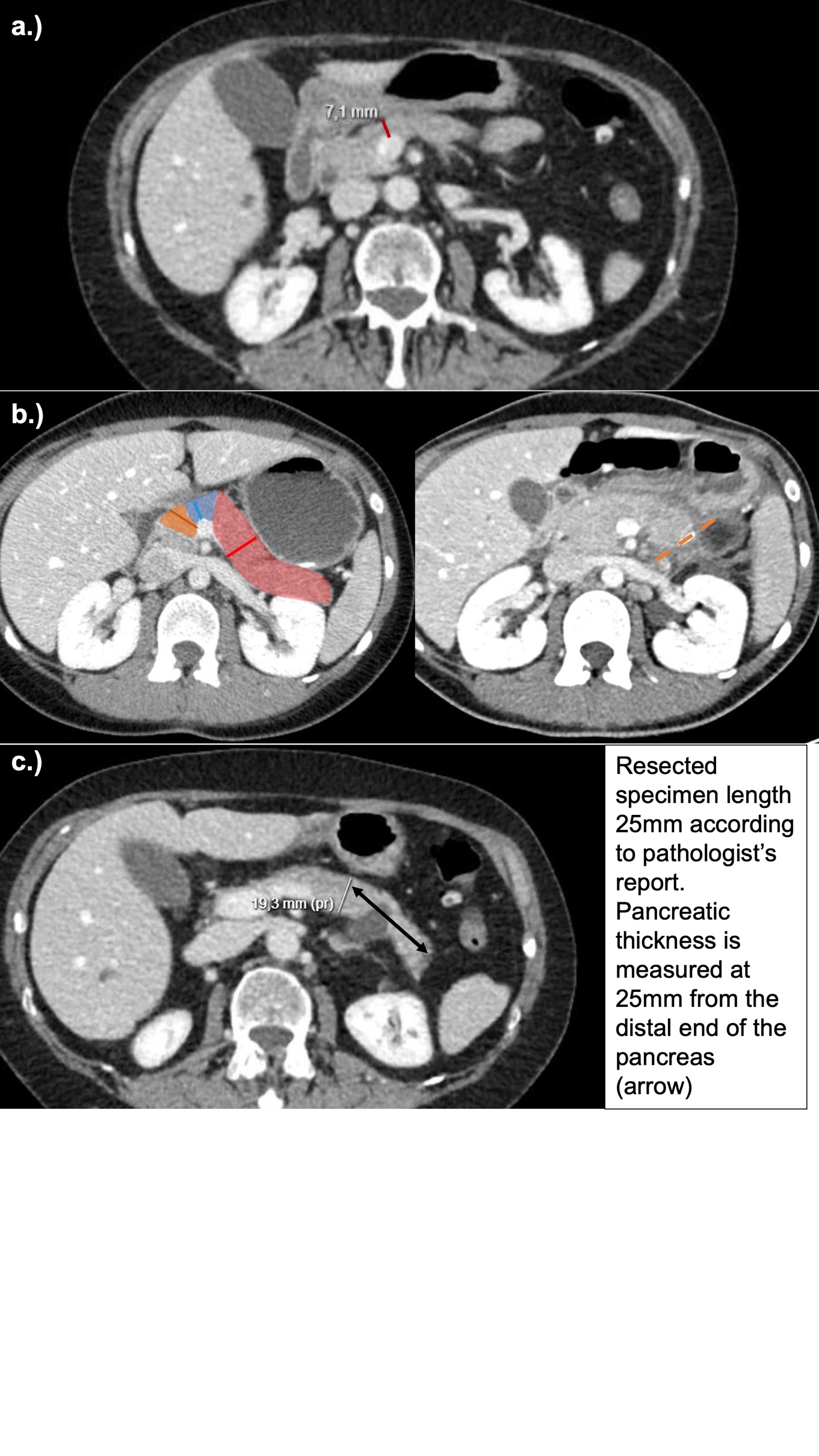


**Supplementary figure 2.** Study flow chart.

**
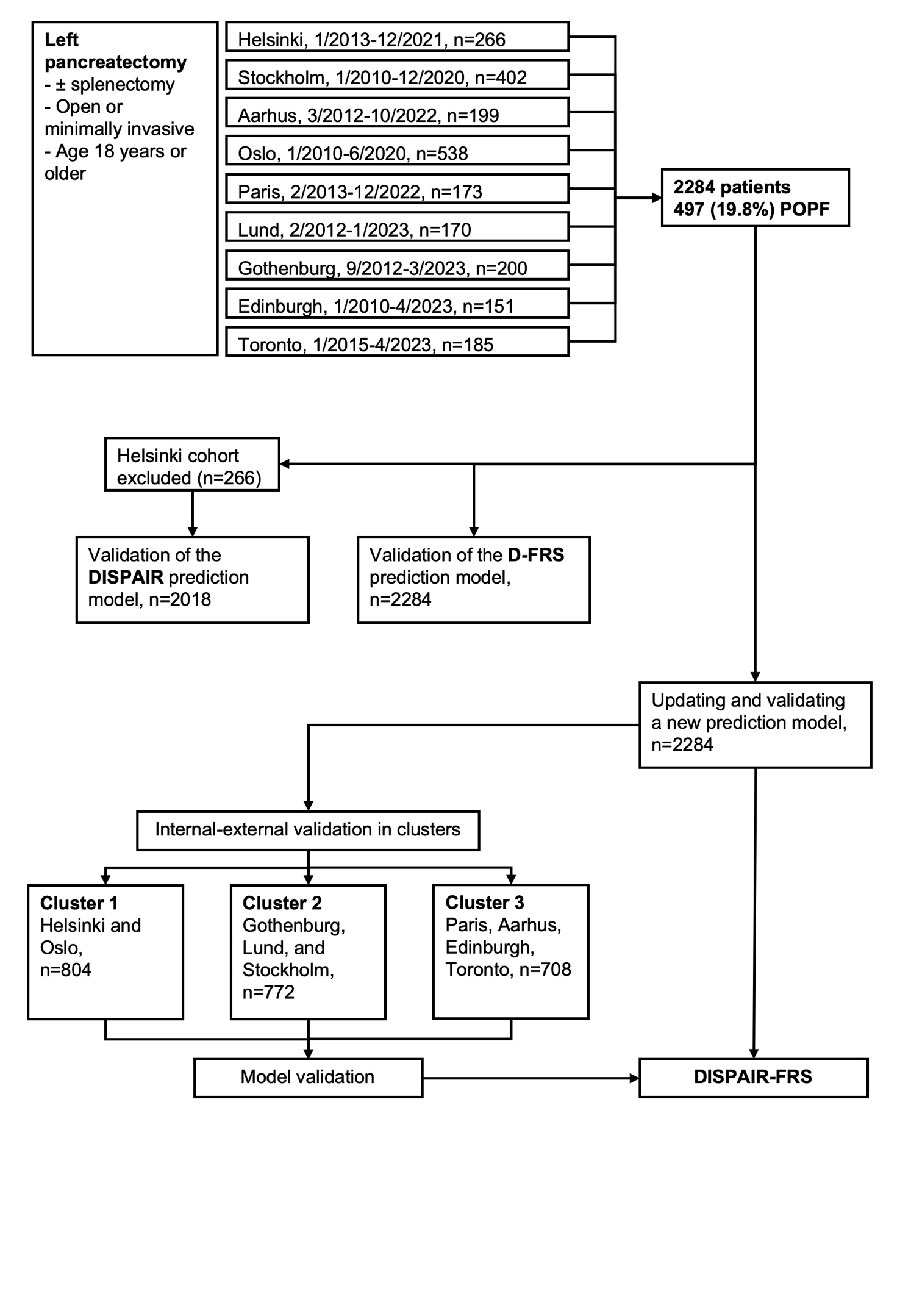
**

Abbreviations: POPF; postoperative pancreatic fistula

**Supplementary figure 3.** Calibration plots and performance metrics for DISPAIR prediction model in each of the participating center. Predicted probability on x-axis, observed probability on y-axis.

**
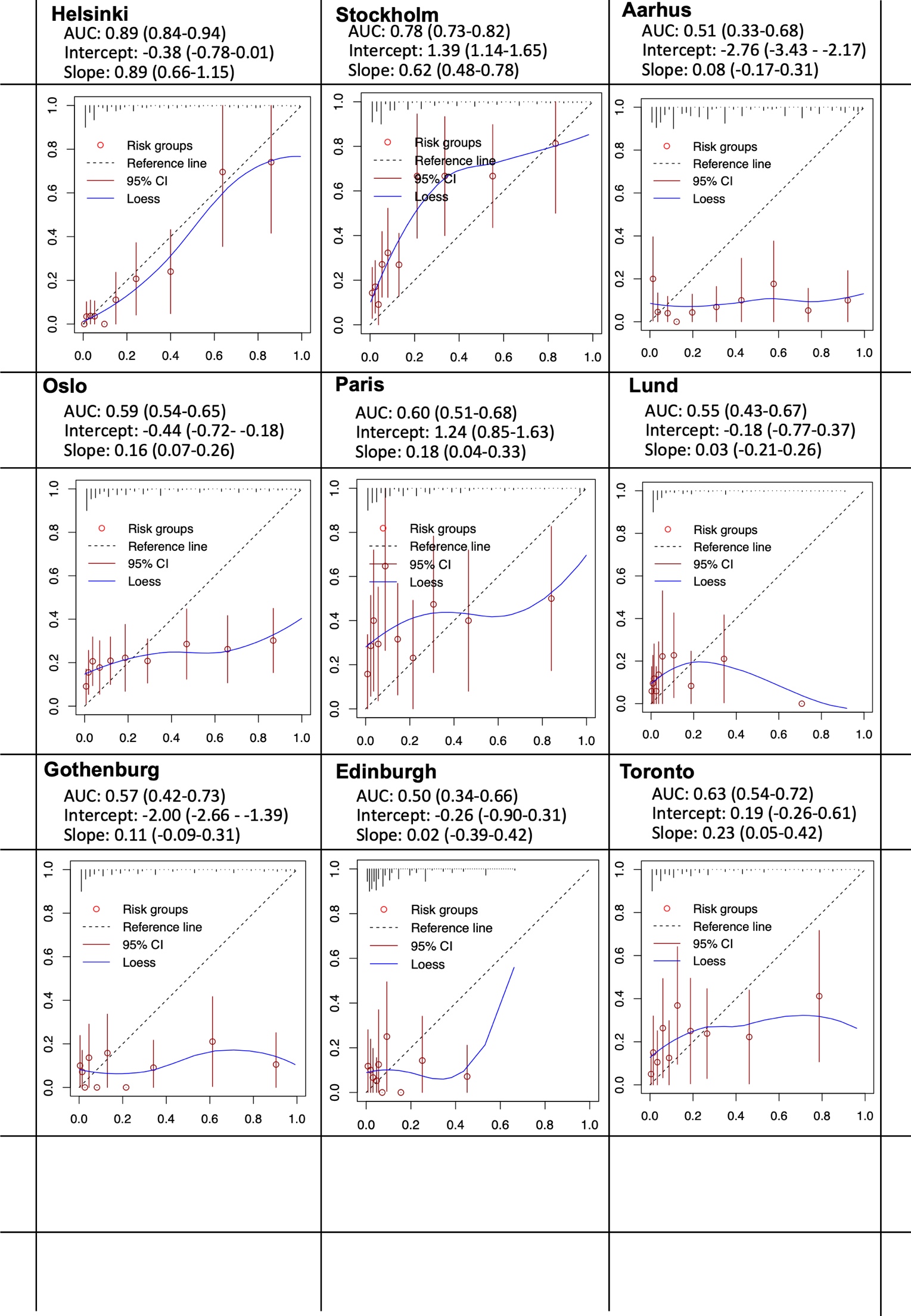
**

Abbreviations: AUC; area under ROC-curve, CITL; calibration in the large = intercept.

Footnote: Red circles (risk groups) are deciles (10% of patients), vertical lines represent distribution of predictions. Loess represents the smoothened calibration curve.

**Supplementary figure 4.** Calibration plots and performance metrics for D-FRS prediction model in each of the participating center. Predicted probability on x-axis, observed probability on y-axis.

**
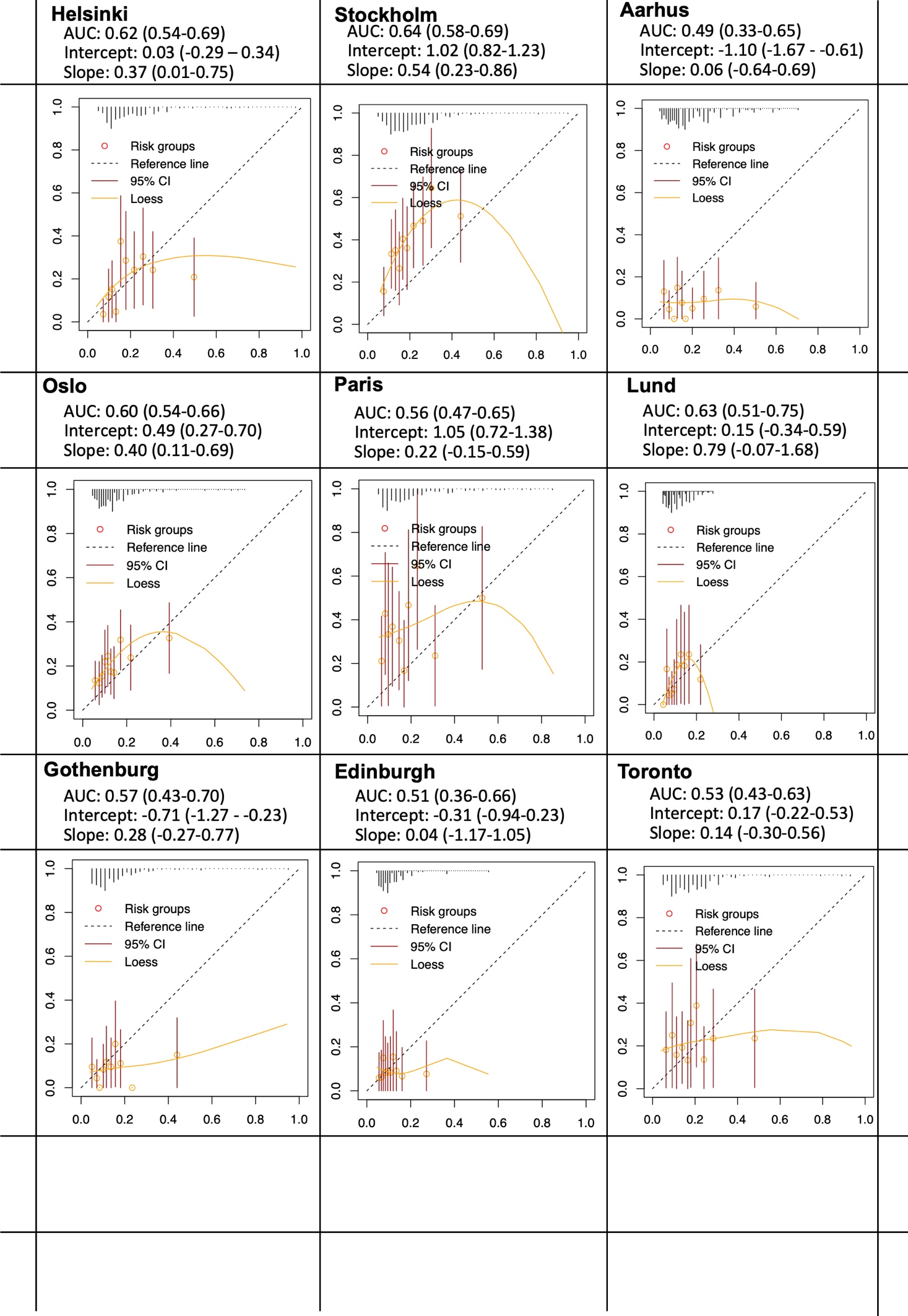
**

Abbreviations: AUC; area under ROC-curve, CITL; calibration in the large = intercept.

Footnote: Red circles (risk groups) are deciles (10% of patients), vertical lines represent distribution of predictions. Loess represents the smoothened calibration curve.

**Supplementary Figure 5.** Association of pancreatic anthropomorphic measurements, age, and BMI to the logit of postoperative pancreatic fistula using splines in 2284 patients undergoing left pancreatectomy.


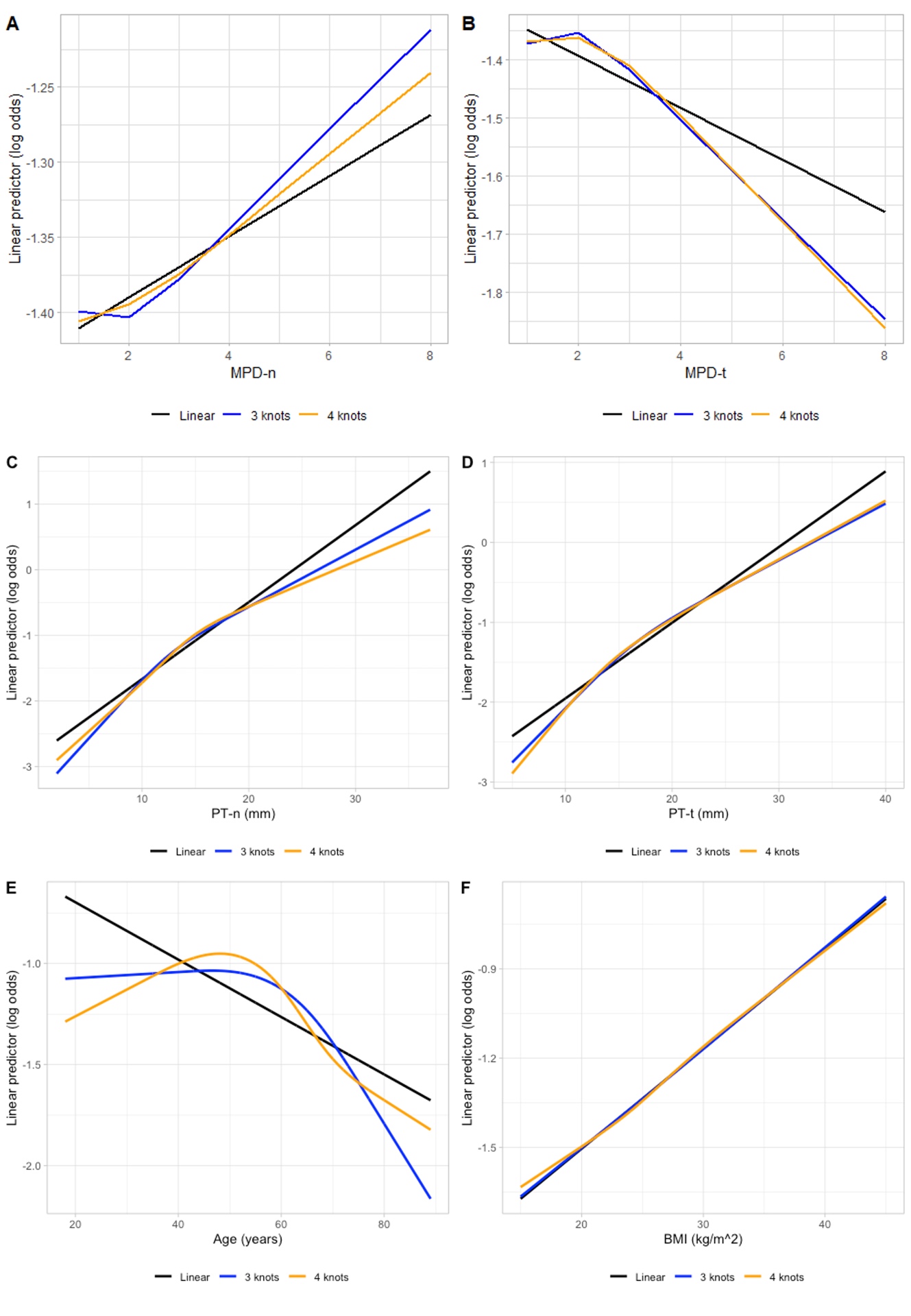


Abbreviations: MPD-n; main pancreatic duct diameter at neck, MPD-t; main pancreatic duct diameter at transection site, PT-n; pancreatic thickness at neck, PT-t; pancreatic thickness at transection site, BMI; body mass index.

**Supplementary Figure 6.** Forest plots for univariable association between **a.)** history of diabetes and **b.)** transection site, and clinically relevant postoperative pancreatic fistula in 2284 patients undergoing left pancreatectomy at nine different centers.


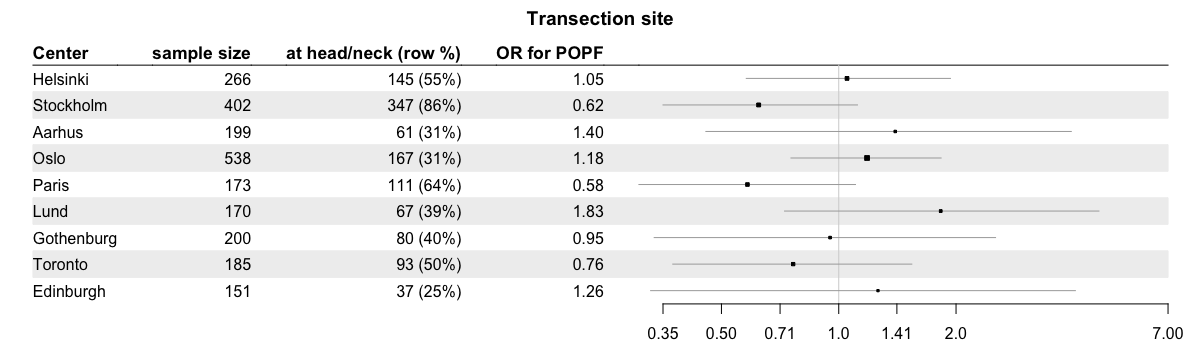

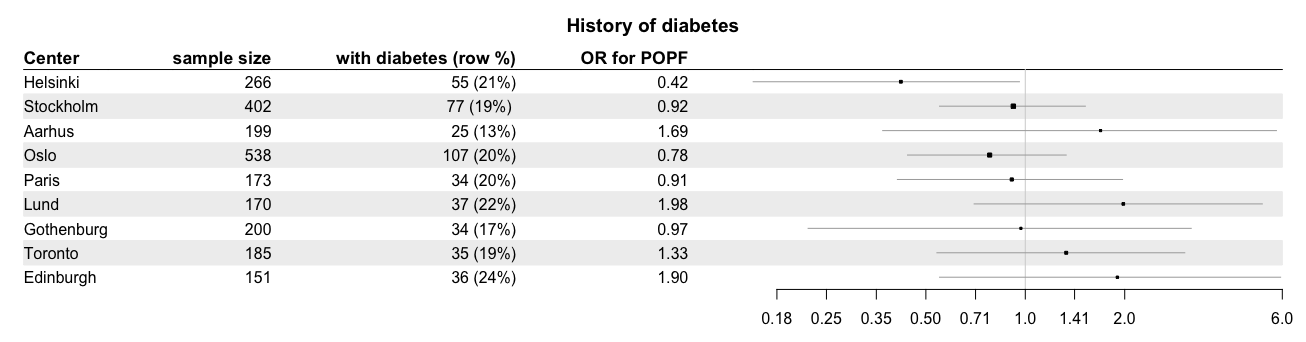
**a)**

**b)**

Performed with multiply imputed data.

Abbreviations: OR; odds ratio, POPF; postoperative pancreatic fistula.

**
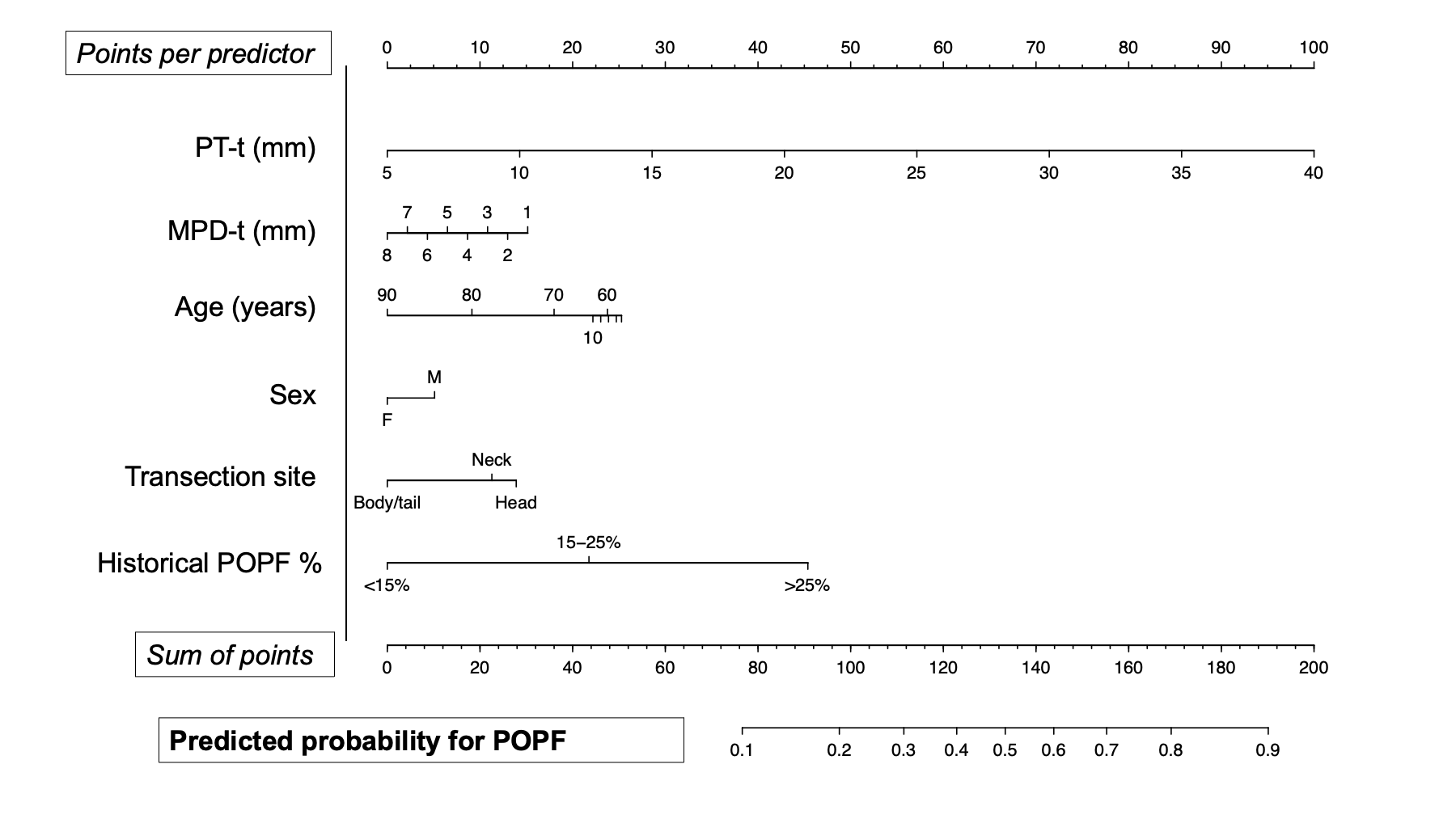
Supplementary figure 7.** Presentation of the DISPAIR-FRS as a nomogram. Link to web-based calculator: [www.tinyurl.com/the-dispair-frs](http://www.tinyurl.com/the-dispair-frs).

Abbreviations: PT-t; pancreatic thickness at transection site, MPD-t; main pancreatic duct diameter at transection site, POPF; clinically relevant postoperative pancreatic fistula.

Footnote: predicted probability for POPF can be calculated from the nomogram by first assessing points per predictor, taking their sum, and finding the corresponding predicted probability from the lowest axis.

**
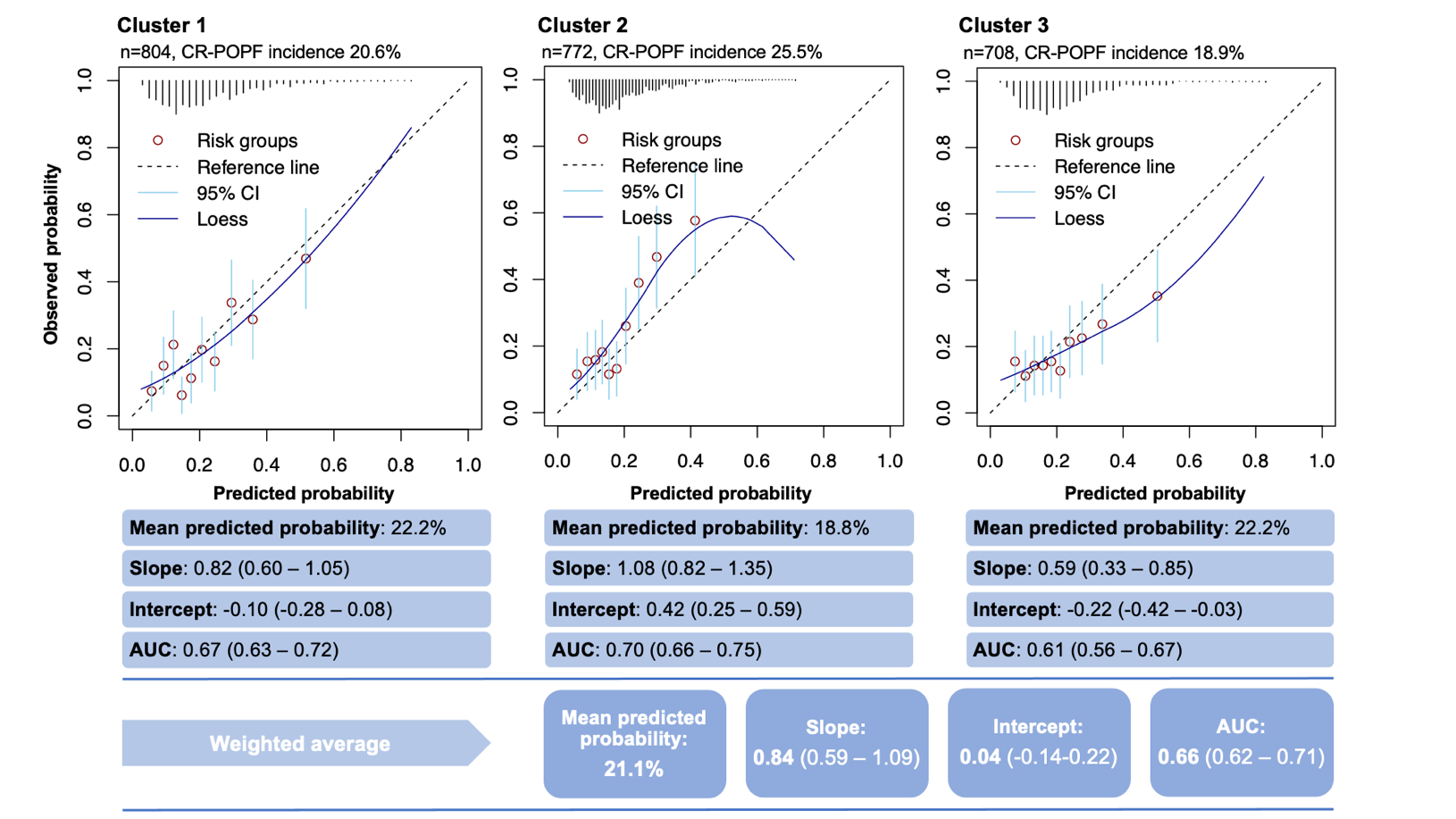
Supplementary Figure 8.** Results of internal-external validation of the DISPAIR-FRS prediction model, but omitting historical POPF incidence, in a cohort of 2284 patients undergoing left pancreatectomy. For each iteration, one cluster was left out for validation and the model was fitted in the remaining clusters.

Abbreviations: POPF; clinically relevant postoperative pancreatic fistula, AUC; area under ROC-curve.

Footnote: In calibration plots red circles represent deciles (10% of patients). Vertical lines represent distribution of predictions (histogram). Centers in cluster 1: Helsinki and Oslo. Centers in cluster 2: Stockholm, Lund, Gothenburg. Centers in cluster 3: Aarhus, Paris, Edinburgh, Toronto.

**Supplementary figure 9.** Histogram of predicted probabilities given by the DISPAIR-FRS prediction model, stratification by postoperative pancreatic fistula. Mean predicted probability with standard deviation (SD) is reported for the total cohort of 2284 left pancreatectomy patients and in patient groups with (n=497 patients) and without (n=1787) postoperative pancreatic fistula.

**
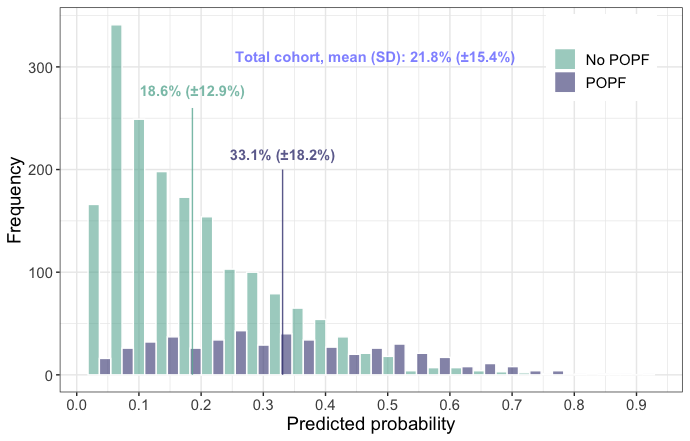
**

Abbreviations: POPF; postoperative pancreatic fistula.

**Supplementary figure 10.** Sensitivity analyses for the full DISPAIR-FRS model. Model performance (calibration plot and AUC-value) has been assessed in different subgroups.

**
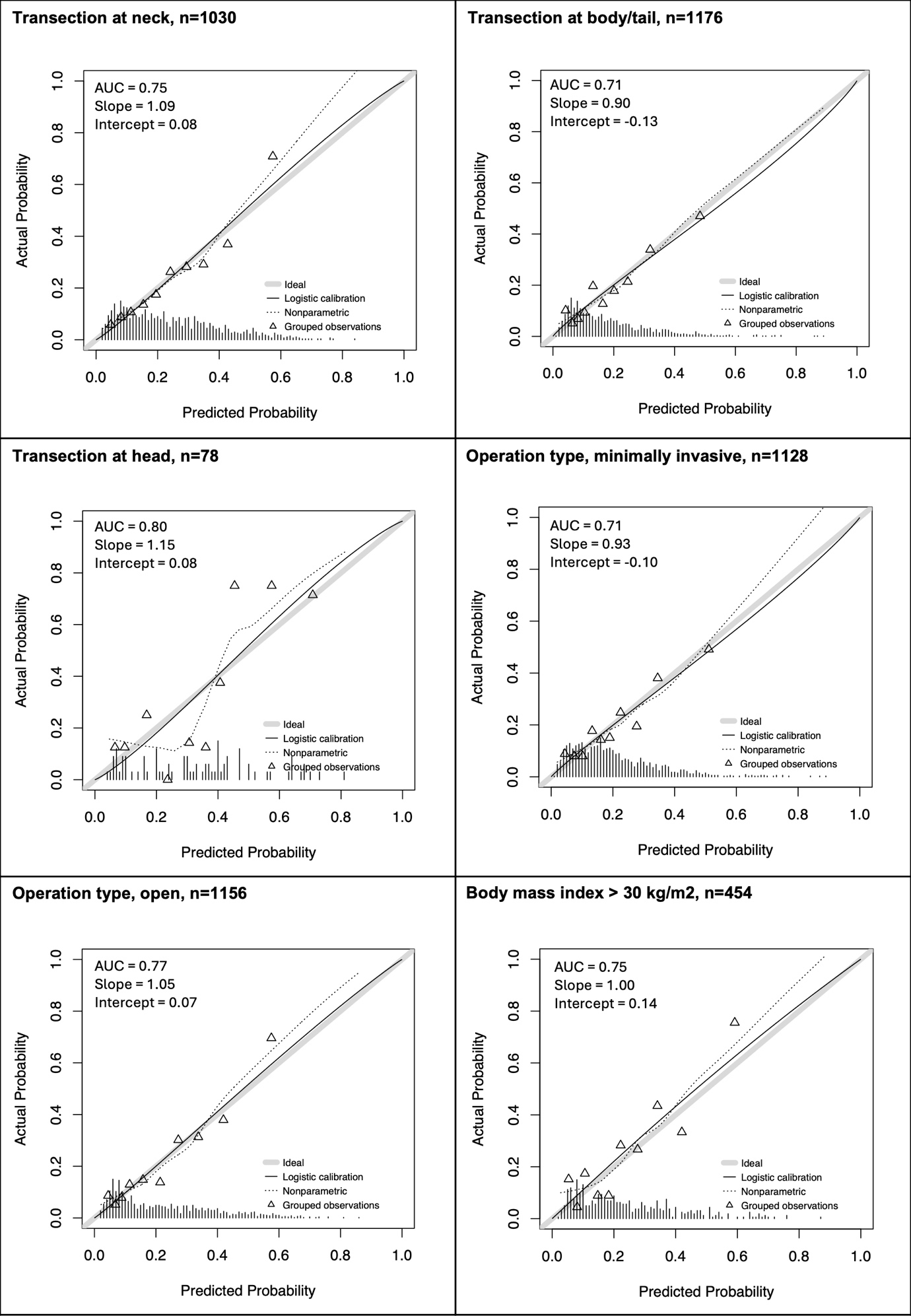
**

**Supplementary figure 10** continued**.**

**
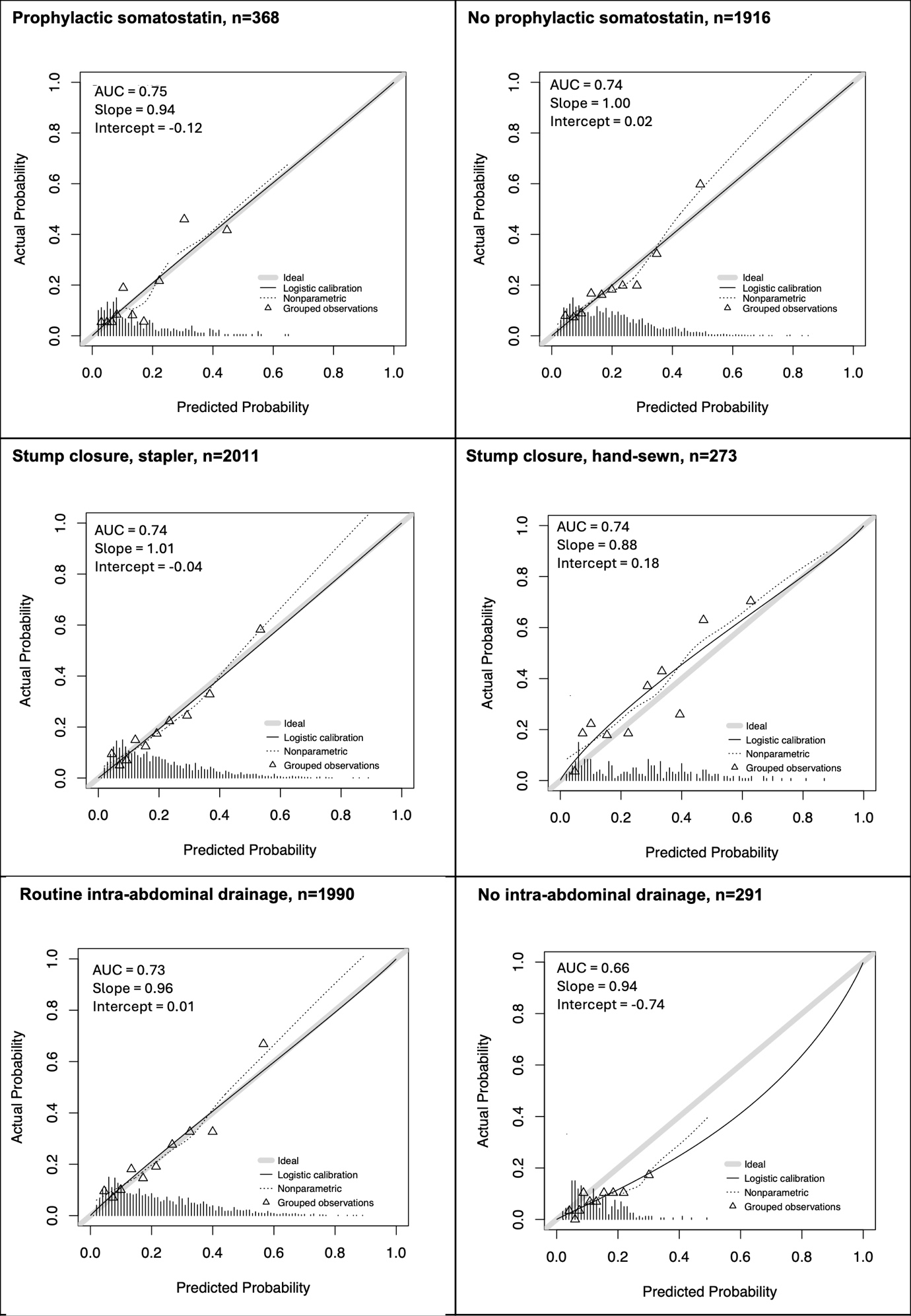
**

**
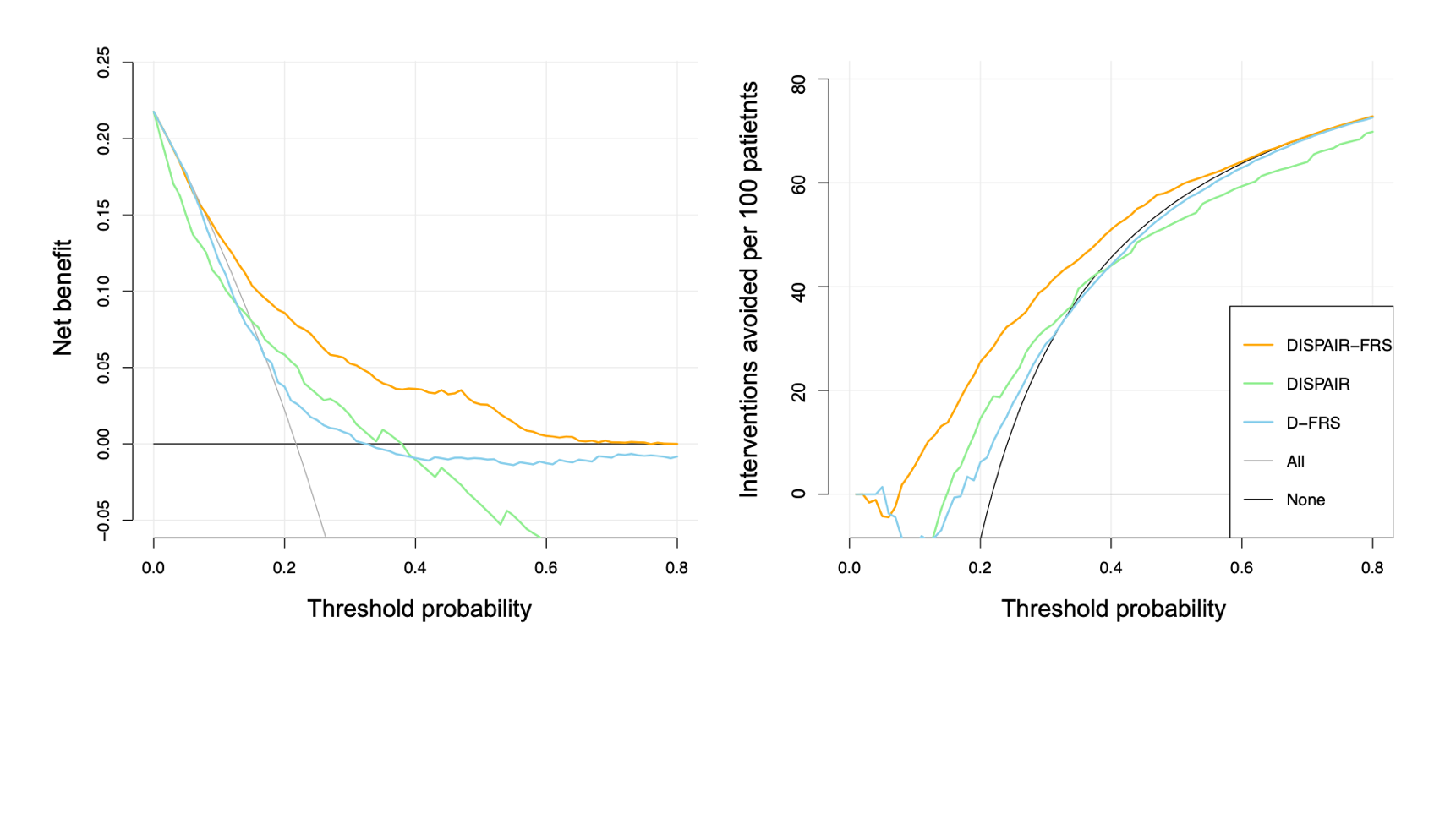
Supplementary figure 11.** Decision-curve analysis for the full DISPAIR-FRS in a cohort of 2284 left pancreatectomy patients, compared to the DISPAIR and the D-FRS.

**Supplementary table 1.** TRIPOD-checklist.

| **Section/Topic** | **Item** |  | **Checklist Item** | **Page** |
| --- | --- | --- | --- | --- |
| **Title and abstract** | | | | |
| Title | 1 | D;V | Identify the study as developing and/or validating a multivariable prediction model, the target population, and the outcome to be predicted. | 1 |
| Abstract | 2 | D;V | Provide a summary of objectives, study design, setting, participants, sample size, predictors, outcome, statistical analysis, results, and conclusions. | 2 |
| **Introduction** | | | | |
| Background and objectives | 3a | D;V | Explain the medical context (including whether diagnostic or prognostic) and rationale for developing or validating the multivariable prediction model, including references to existing models. | 4 |
|  | 3b | D;V | Specify the objectives, including whether the study describes the development or validation of the model or both. | 4 |
| **Methods** | | | | |
| Source of data | 4a | D;V | Describe the study design or source of data (e.g., randomized trial, cohort, or registry data), separately for the development and validation data sets, if applicable. | 4 |
|  | 4b | D;V | Specify the key study dates, including start of accrual; end of accrual; and, if applicable, end of follow-up. | 4 |
| Participants | 5a | D;V | Specify key elements of the study setting (e.g., primary care, secondary care, general population) including number and location of centres. | 4 |
|  | 5b | D;V | Describe eligibility criteria for participants. | 4 |
|  | 5c | D;V | Give details of treatments received, if relevant. | 4 |
| Outcome | 6a | D;V | Clearly define the outcome that is predicted by the prediction model, including how and when assessed. | 5 |
|  | 6b | D;V | Report any actions to blind assessment of the outcome to be predicted. | - |
| Predictors | 7a | D;V | Clearly define all predictors used in developing or validating the multivariable prediction model, including how and when they were measured. | 5 |
|  | 7b | D;V | Report any actions to blind assessment of predictors for the outcome and other predictors. | - |
| Sample size | 8 | D;V | Explain how the study size was arrived at. | 5 |
| Missing data | 9 | D;V | Describe how missing data were handled (e.g., complete-case analysis, single imputation, multiple imputation) with details of any imputation method. | 5 |
| Statistical analysis methods | 10a | D | Describe how predictors were handled in the analyses. | 5 |
|  | 10b | D | Specify type of model, all model-building procedures (including any predictor selection), and method for internal validation. | 6 |
|  | 10c | V | For validation, describe how the predictions were calculated. | 5 |
|  | 10d | D;V | Specify all measures used to assess model performance and, if relevant, to compare multiple models. | 6 |
|  | 10e | V | Describe any model updating (e.g., recalibration) arising from the validation, if done. | 6 |
| Risk groups | 11 | D;V | Provide details on how risk groups were created, if done. | - |
| Development vs. validation | 12 | V | For validation, identify any differences from the development data in setting, eligibility criteria, outcome, and predictors. | - |
| **Results** | | | | |
| Participants | 13a | D;V | Describe the flow of participants through the study, including the number of participants with and without the outcome and, if applicable, a summary of the follow-up time. A diagram may be helpful. | 7 |
|  | 13b | D;V | Describe the characteristics of the participants (basic demographics, clinical features, available predictors), including the number of participants with missing data for predictors and outcome. | Table 1 |
|  | 13c | V | For validation, show a comparison with the development data of the distribution of important variables (demographics, predictors and outcome). | - |
| Model development | 14a | D | Specify the number of participants and outcome events in each analysis. | 8 |
|  | 14b | D | If done, report the unadjusted association between each candidate predictor and outcome. | 8 |
| Model specification | 15a | D | Present the full prediction model to allow predictions for individuals (i.e., all regression coefficients, and model intercept or baseline survival at a given time point). | Table 2, page 9 |
|  | 15b | D | Explain how to the use the prediction model. | 9 |
| Model performance | 16 | D;V | Report performance measures (with CIs) for the prediction model. | Figure 3, Supplementary figure 8, page 9 |
| Model-updating | 17 | V | If done, report the results from any model updating (i.e., model specification, model performance). | - |
| **Discussion** | | | | |
| Limitations | 18 | D;V | Discuss any limitations of the study (such as nonrepresentative sample, few events per predictor, missing data). | 11 |
| Interpretation | 19a | V | For validation, discuss the results with reference to performance in the development data, and any other validation data. | 10 |
|  | 19b | D;V | Give an overall interpretation of the results, considering objectives, limitations, results from similar studies, and other relevant evidence. | 9-11 |
| Implications | 20 | D;V | Discuss the potential clinical use of the model and implications for future research. | 11 |
| **Other information** | | | | |
| Supplementary information | 21 | D;V | Provide information about the availability of supplementary resources, such as study protocol, Web calculator, and data sets. | 9 |
| Funding | 22 | D;V | Give the source of funding and the role of the funders for the present study. | 12 |

|  | | **Helsinki** | **Stockholm** | **Aarhus** | **Oslo** | **Paris** | **Lund** | **Gothenburg** | **Edinburgh** | **Toronto** |
| --- | --- | --- | --- | --- | --- | --- | --- | --- | --- | --- |
| ***Time span*** | | ***1/2013-12/2021*** | ***1/2010-12/2020*** | ***3/2012-10/2022*** | ***1/2010-6/2020*** | ***2/2013-12/2022*** | ***2/2012-1/2023*** | ***9/2012-3/2023*** | ***1/2010-4/2023*** | ***1/2015-4/2023*** |
| ***Sample size, n (%)*** | | *266 (11.6%)* | *402 (17.6%)* | *199 (8.7%)* | *538 (23.6%)* | *173 (7.6%)* | *170 (7.4%)* | *200 (8.8%)* | *151 (6.6%)* | *185 (8.1%)* |
| **Age, median (IQR)** | | 65 (54-71) | 67 (56-73) | 63 (51-70) | 65 (54-72) | 63 (50-72) | 68 (56-74) | 69 (55-75) | 61 (48-69) | 64 (54-71) |
| **Male sex, n (%)** | | 107 (40.2%) | 183 (45.5%) | 96 (48.2%) | 281 (52.2%) | 91 (52.6%) | 66 (38.8%) | 86 (43.0%) | 62 (41.1%) | 86 (46.5%) |
| **BMI, median (IQR)** | | 26.2 (23.8-29.8) | 25.8 (22.7-29.3) | 26.0 (22.9-29.4) | 25.5 (22.8-28.6) | 25.0 (22.0-28.0) | 25.3 (22.8-28.0) | 27.0 (23.0-30.0) | 26.3 (22.6-31.0) | 27.0 (23.2-30.9) |
| **History of myocardial infarction** | | 11 (4.1%) | 29 (7.2%) | 4 (2.0%) | 33 (6.1%) | 13 (7.5%) | 12 (7.1%) | 10 (5.0%) | 16 (10.5%) | 2 (1.1%) |
| **History of COPD** | | 41 (15.4%) | 41 (10.2%) | 6 (3.0%) | 85 (15.8%) | 15 (8.6%) | 16 (9.4%) | 2 (1.0%) | 14 (9.2%) | 4 (2.2%) |
| **History of diabetes** | | 55 (20.7%) | 77 (19.2%) | 26 (13.0%) | 107 (19.9%) | 34 (19.5%) | 37 (21.8%) | 34 (17.0%) | 36 (23.7%) | 35 (18.9%) |
| **Neoadjuvant therapy (any), n (%)** | | 32 (12.0%) | 12 (3.0%) | 13 (6.5%) | 13 (2.4%) | 28 (16.1%) | 9 (5.3%) | 11 (5.5%) | 8 (5.3%) | 13 (7.0%) |
| **Operation type** | Open | 118 (44.4%) | 337 (83.8%) | 176 (88.4%) | 38 (7.1%) | 110 (63.6%) | 102 (60.0%) | 72 (36.0%) | 111 (73.0%) | 91 (49.2%) |
|  | Lap | 113 (42.5% | 43 (10.7%) | 23 (11.5%) | 500 (93.1%) | 62 (35.6%) | 61 (35.9%) | 110 (55.0%) | 41 (27.0%) | 93 (50.3%) |
|  | Rob | 34 (12.8%) | 22 (5.5%) | 0 | 0 | 1 (0.6%) | 7 (4.1%) | 17 (8.5%) | 0 | 0 |
| **Transection method** | Stapler | 226 (85.0%) | 330 (82.1%) | 188 (94.0%) | 537 (99.8%) | 96 (55.5%) | 119 (70.0%) | 194 (97.0%) | 125 (82.8%) | 165 (89.2%) |
|  | Hand-sewn | 40 (15.0%) | 71 (17.7%) | 3 (1.5%) | 0 | 74 (42.8%) | 51 (30.0%) | 3 (1.5%) | 26 (17.2%) | 0 |
|  | Other | 0 | 0 | 6 (3.0%) | 1 (0.2%) | 0 | 0 | 3 (1.5%) | 0 | 20 (10.8%) |
| **Transection site** | Head | 12 (4.5%) | 31 (7.7%) | 0 | 0 | 12 (6.9%) | 6 (3.5%) | 3 (1.5%) | 1 (0.7%) | 2 (1.1%) |
|  | Neck | 133 (50.0%) | 292 (72.6%) | 58 (29.1%) | 166 (30.9%) | 86 (49.7%) | 56 (32.9%) | 77 (38.5%) | 8 (5.3%) | 91 (49.2%) |
|  | Body/tail | 121 (45.5%) | 47 (11.7%) | 128 (64.3%) | 369 (68.6%) | 58 (33.5%) | 98 (57.6%) | 120 (60.0%) | 35 (23.2%) | 92 (49.7%) |
| **Use of somatostatin analogue** | None | 123 (46.2%) | 293 (72.9%) | 198 (99.5%) | 530 (98.5%) | 107 (61.8%) | 6 (3.5%) | 192 (96.0%) | 142 (94.0%) | 176 (95.1%) |
|  | Prophylactic | 143 (53.8%) | 0 | 0 | 0 | 54 (31.2%) | 160 (94.1%) | 7 (3.5%) | 5 (3.4%) | 3 (1.6%) |
|  | Treatment | 0 | 109 (27.1%) | 1 (0.5%) | 8 (1.5%) | 12 (6.9%) | 4 (2.4%) | 1 (0.5%) | 0 | 6 (3.2%) |
| **Perioperative intra-abdominal drainage** | | 266 (100%) | 402 (100%) | 44 (22.1%) | 538 (100%) | 170 (98.3%) | 142 (83.5%) | 199 (99.5%) | 138 (91.4%) | 92 (49.7%) |
| **Initial length of stay (d), median (IQR)** | | 6 (5-8) | 9 (7-14) | 7 (5-9) | 5 (4-8) | 20 (13-30) | 7 (6-9) | 9 (7-14) | 7 (6-9) | 5 (4-8) |
| **Readmissions, n (%)** | | 45 (16.9%) | 91 (22.6%) | 22 (11.1%) | 61 (11.3%) | 34 (19.7%) | 17 (10.0%) | 28 (14.0%) | 9 (6.0%) | 36 (19.5%) |
| **90-day mortality, n (%)** | | 2 (0.8%) | 8 (2.0%) | 6 (3.0%) | 4 (0.7%) | 6 (3.5%) | 0 | 1 (0.5%) | 3 (2.0%) | 4 (2.2%) |
| **POPF (grade B/C), n (%), up to 30 POD** | | 54 (20.3%) | 158 (39.3%) | 16 (8.0%) | 112 (20.8%) | 64 (37.0%) | 21 (12.4%) | 18 (9.0%) | 14 (9.3%) | 40 (21.6%) |
| **Tumor histology / pathologist’s report** | PDAC | 69 (25.9%) | 104 (25.9%) | 59 (29.6%) | 146 (27.1%) | 70 (40.5%) | 61 (35.9%) | 55 (27.5%) | 26 (17.2%) | 60 (32.4%) |
|  | IPMN | 28 (10.5%) | 97 (24.1%) | 6 (3.0%) | 53 (9.9%) | 15 (8.7%) | 37 (21.8%) | 31 (15.5%) | 16 (10.6%) | 13 (7.0%) |
|  | NET | 78 (29.3%) | 75 (18.7%) | 66 (33.2%) | 144 (26.8%) | 41 (23.7%) | 27 (15.9%) | 59 (29.5%) | 41 (27.2%) | 53 (28.6%) |
|  | Other malign | 22 (8.3%) | 31 (7.7%) | 16 (8.0%) | 45 (8.4%) | 15 (8.7%) | 14 (8.2%) | 7 (3.5%) | 27 (17.9%) | 15 (8.1%) |
|  | Other benign | 56 (21.1%) | 95 (23.6%) | 40 (20.1%) | 140 (26.0%) | 32 (18.5%) | 30 (17.6%) | 43 (21.5%) | 31 (20.5%) | 38 (20.5%) |
|  | Dysplasia | 6 (2.3%) | 0 | 0 | 1 (0.2%) | 0 | 1 (0.6%) | 3 (1.5%) | 1 (0.7%) | 4 (2.2%) |
|  | Not diagnostic | 2 (0.8%) | 0 | 12 (6.0%) | 9 (1.7%) | 0 | 0 | 2 (1.0%) | 9 (6.0%) | 2 (1.1%) |

**Supplementary table 2.** Basic demographics, perioperative interventions and outcomes in a cohort of 2284 patients undergoing left pancreatectomy, data presented center-wise.

Abbreviations: COPD; chronic obstructive pulmonary disease, POPF; postoperative pancreatic fistula, POD; postoperative day.

**Supplementary table 3.** Different clinical outcomes stratified by POPF risk given by the DISPAIR-FRS in 2284 patients undergoing left pancreatectomy.

| **Outcome** | **Low risk (<10%)**  **n=627** | **Moderate risk (10-30%)**  **n=1064** | **High risk (>30%)**  **n=593** | ***P*** |
| --- | --- | --- | --- | --- |
| **POPF, grade B/C** | 50 (8.0%) | 185 (17.4%) | 262 (44.2%) | <0.001 |
| **POPF, grade B** | 48 (7.7%) | 171 (16.1%) | 238 (40.1%) | <0.001 |
| **POPF, grade C** | 2 (0.3%) | 14 (1.3%) | 24 (4.0%) | <0.001 |
| **PPH, grade B/C** | 15 (2.4%) | 50 (4.7%) | 45 (7.6%) | <0.001 |
| **Reoperation** | 22 (3.5%) | 57 (5.4%) | 59 (9.9%) | <0.001 |
| **Readmission** | 67 (10.7%) | 144 (13.5%) | 142 (23.9%) | <0.001 |
| **90-day mortality** | 10 (1.6%) | 14 (1.3%) | 10 (1.7%) | 0.809 |
| **Length of stay (d)** | 7 (6-10) | 6 (5-9) | 10 (7-16) | <0.001 |

Abbreviations: POPF; postoperative pancreatic fistula, PPH; postpancreatectomy hemorrhage.
